# Simultaneous Measurement of Viscosity and Optical Density of Bacterial Growth and Death in a Microdroplet

**DOI:** 10.3390/mi9050251

**Published:** 2018-05-21

**Authors:** Karolina Sklodowska, Pawel R. Debski, Jacek A. Michalski, Piotr M. Korczyk, Miroslaw Dolata, Miroslaw Zajac, Slawomir Jakiela

**Affiliations:** 1 Department of Biophysics, Warsaw University of Life Sciences, 159 Nowoursynowska Street, 02776 Warsaw, Poland; karolina_sklodowska@sggw.pl (K.S.); pawel_debski@sggw.pl (P.R.D.); miroslaw_zajac@sggw.pl (M.Z.); 2 Department of Plant Genetics, Breeding and Biotechnology, Warsaw University of Life Sciences, 159 Nowoursynowska Street, 02776 Warsaw, Poland; 3 Faculty of Civil Engineering, Mechanics and Petrochemistry, Warsaw University of Technology, 17 Lukasiewicza Street, 09400 Plock, Poland; jacek.michalski@pw.edu.pl; 4 Institute of Fundamental Technological Research, Polish Academy of Sciences, Pawinskiego 5B, 02106 Warsaw, Poland; piotr.korczyk@ippt.pan.pl; 5 Department of Econophysics and Physics Application, Warsaw University of Life Sciences, 159 Nowoursynowska Street, 02776 Warsaw, Poland; miroslaw_dolata@sggw.pl

**Keywords:** droplet microfluidics, cell growth, viscosity, rheology, *Escherichia coli*

## Abstract

Herein, we describe a novel method for the assessment of droplet viscosity moving inside microfluidic channels. The method allows for the monitoring of the rate of the continuous growth of bacterial culture. It is based on the analysis of the hydrodynamic resistance of a droplet that is present in a microfluidic channel, which affects its motion. As a result, we were able to observe and quantify the change in the viscosity of the dispersed phase that is caused by the increasing population of interacting bacteria inside a size-limited system. The technique allows for finding the correlation between the viscosity of the medium with a bacterial culture and its optical density. These features, together with the high precision of the measurement, make our viscometer a promising tool for various experiments in the field of analytical chemistry and microbiology, where the rigorous control of the conditions of the reaction and the monitoring of the size of bacterial culture are vital.

## 1. Introduction

From the physical point of view, every molecule that is suspended in a fluid influences the fluid viscosity as a result of the hydrodynamic interactions [1]. For this reason, the study of the hydrodynamic properties of a medium, and particularly its viscosity, should provide information on the behaviour of biological organisms, as their proliferation influences the viscosity of the medium. In this respect, microfluidics is the most promising technology for measuring the mechanical properties of Biosystems, since its capabilities are nowadays widely explored and described [2,3]. In addition, the ability to measure the viscosity of chemical systems that change their shapes as a result of chemical factors [4,5], is also attractive.

Classical viscometers, such as falling ball [6] (Hoeppler), cone-and-plate [7], rotating disc [8], and U-tube capillaries (e.g., Ostwald [9] or Ubbelohde [10]), despite some drawbacks (repetitive tests are required, large volume consumptions, and complex cleaning procedure [11]), are still used to measure the various fluids in the medical and biological fields. Moreover, optical technology also enables viscosity measurements with the use of optical tweezers [12], photoacoustic [13], and fluorescence [14] methods. However, current microfluidic rheometers have stimulated the interest of many engineers and scientists, primarily because of the counter-intuitive behaviour of the multi-phase fluid flow in microscale channels [15,16]. One approach constitutes a liquid sensing system, with electrodes that are located inside the microfluidic channels, allowing for the measurement of the viscosity of nanoliter droplets. The operation of the system is based on the consecutive measurements of the liquid that is passing through the wires, as well as of the convective loss of heat of a flowing droplet [17]. Furthermore, a parallel microfluidic circuit allows for the comparison of an unknown hydraulic resistance of one branch, with a known resistance of the second microchannel [5,18]. Another approach measures the viscosity of droplets using the tracking of particles inside the water phase with a camera [19]. In turn, Livak-Dahl et al. [20] has calculated the droplet viscosity using an abrupt channel constriction, which has been characterised by a high hydrodynamic resistance, compared to the whole microfluidic system. A different solution has been proposed by Li et al. [21]. The authors took advantage of a relationship between the viscosity and the length of the generated droplets in a flow-focusing microfluidic junction. However, in the majority of the technical solutions, a high-speed camera and advanced image analysis are required so as to provide reliable outcomes.

The earliest viscosity measurement of the bacterial culture was presented by Jacques Bronfenbrenner [22]. However, as shown recently by Leal’s group [23,24], the viscosity of a bacteria population exhibits non-intuitive alternating behaviour during the exponential growth phase, which depends strongly on the strain type. It was reported that in highly viscous bacterial mixtures, the bacterial mobility is affected and that the drag forces during their swimming are increased [25,26]. Furthermore, recent studies have indicated that increasing of the viscosity of the medium during the biofilm formation may allow the bacteria to tolerate higher concentrations of antibiotics [27]. Moreover, in the high viscosity populations of bacteria, the intercellular cooperation is promoted [28].

Herein, we propose a new system that measures, simultaneously and in real-time manner, the viscosity and light absorbance of fluid that has been introduced to a simple microfluidic chip as a small droplet. Although the presented approach is not optimised for high throughput and the shortest turnaround time, the device we show is simple and requires only a limited laboratory equipment, while providing measurements that are based solely on the fundamental physical parameters for the flow of a single droplet. Instead of a strong and complex mathematical model in the image analysis, we propose a system consisting two simple optical sensors placed at the capillary to measure the passage time of a droplet between them. Hence, knowing the relation of droplet viscosity vs. passage time (calibration required), we can quickly calculate the viscosity of the measured solution in a droplet.

## 2. Materials and Methods

### 2.1. Reagents

The continuous phase was hydrofluoroether (HFE)-7200 (Novec 7200 Engineering Fluid, 3M Company, St. Paul, MN, USA) with 5% *w*/*w* of 1H,1H,2H,2H-Perfluoroctanol (Sigma-Aldrich, Steinheim, Germany), and had a viscosity of *μ_oil_* = 0.395 cP [29] (1 cP = 1 mPa·s) at 37 °C and an oxygen solubility above 100 mL of gas per 1 L of liquid at 1 bar of air pressure at 35 °C [30].

A Luria-Bertani Broth (LB Broth, Biocorp, Poland) was used in all of the experiments. As a model organism we used *Escherichia coli* strain DH5α (ThermoFisher Scientific, catalogue number 18263012, Waltham, MA, USA). The prepared stock solution of the cells in the LB broth, which contained 30% glycerol (Sigma-Aldrich, Poznań, Poland), was stored at −80 °C. Before the experiments, the cells were streaked on LB agar plates and were incubated overnight. We picked individual colonies, used them to inoculate liquid LB broth, cultured the cells at 37 °C with shaking at 200 rpm overnight, transferred aliquots to the fresh LB media, and grew them until the absorbance at 600 nm reached 0.5. The culture was diluted tenfold in a fresh medium before the suspensions of *Escherichia coli* were transferred into the microfluidic system. Each experiment was repeated ten times.

The interfacial tension between the LB medium and HFE 7200 fluid, with 5% *w*/*w* Perfluoroctanol, was 19 ± 0.5 mN/m, which was measured using the pending droplet method.

The calibration of the viscosity measurement was performed using various mixtures of water and glycerol. Prior to the calibration of the device, the viscosity of solutions were measured using a rolling-ball viscometer (Minivis 445, Grabner Instruments, Vienna, Austria). Our device could generate monodisperse droplets up to a viscosity that was equal to 50 cP. The repetitive formation of the droplets in the t-junction was problematic for the droplets that were characterized by the viscosity larger than 60 cP.

### 2.2. Requirements for the Microfludic Chip-Method Description

In some publications, a stable flow of the continuous phase was established with the use of a long, thin capillary between the pressurised tank and the microfluidic system [31,32]. This approach enabled the assessment of the change of speed of the water phase, relative to the constant flow rate of the oil phase. However, more precise results were obtained while using a microfluidic system that provided a constant difference of pressure that was applied between the input (e.g., the continuous phase tank) and output (e.g., the waste tank) to/from the chip [33]. Hence, from analogically to electronic circuits to the microfluidic systems, we assumed that the hydrodynamic resistance of the channel that was filled with oil was $R_{c}$ and that the hydrodynamic resistance of the droplet with a constant volume was $R_{d}$. For our device, we could express, based on the Kirchhoff laws, that Rc+Rd=ΔpQ=ΔpSv≅ΔtΔpSl, where Δp was the pressure difference between the input and output of the system, Q was the volumetric flow rate, S was the surface of a cross-section of the tubing, v was the average linear speed of the oil, which was transferred to the speed of a droplet, l was the distance between the sensors, and Δt was the passage time of a droplet between sensors. In turn, from the Hagen–Pouiseuille law for a long droplet, we could approximate that $R_{d}\sim \mu_{d}$. The hydrodynamic resistance of the channel that was filled with oil was persistent all times during the measurements. Therefore, all of the changes in the viscosity of a droplet should have been linear in its passage time between sensors in the first approximation. As a result, the droplet flowed faster when its viscosity decreased and slowed down when its viscosity rose [33,34]. Therefore, the growth of bacteria culture in droplets should have led to the change in the viscosity that was detected by the measuring system.

The design of the microfluidic system that was aimed at studying the properties of the viscous droplets should be characterized by the following: (i) low hydrodynamic resistance of all of the leads to the main measuring channel, (ii) possibility of reversing the flow in order to circulate a droplet back and forth, (iii) continuous and real-time monitoring of droplets, and (iv) changeable and adjustable pressure to force the flow of the continuous (oil) phase (in this case, the use of pumps facilitating steady flow is inadvisable). Among these, the condition (i) was crucial for the presented measurement method. To achieve the high accuracy and precision of the measurement of the droplet viscosity, one needed to maintain a favourable ratio of the hydrodynamic resistances of the droplet to the whole microfluidic system. Herein, for a 2.4-µL droplet that contained a LB broth medium, this ratio was ~1:500 for the capillary numbers Ca = *μ_o_*·*v*/*γ* ~ 2 × 10^−4^ (*μ_o_*—dynamic viscosity of the oil, *v*—the speed of the droplet, and *γ*—the interfacial tension between oil and droplet). All of the channels that supplied fluids to the chip should have had a relatively large cross-section so as to reduce the additional hydrodynamic resistance that they add to the system. Another challenge was the choice of the continuous phase fluid which should have been characterised by a low viscosity and high oxygen solubility because of the need for incubating bacteria under aerobic conditions.

### 2.3. Construction of the Microfluidic System

**Figure 1:**
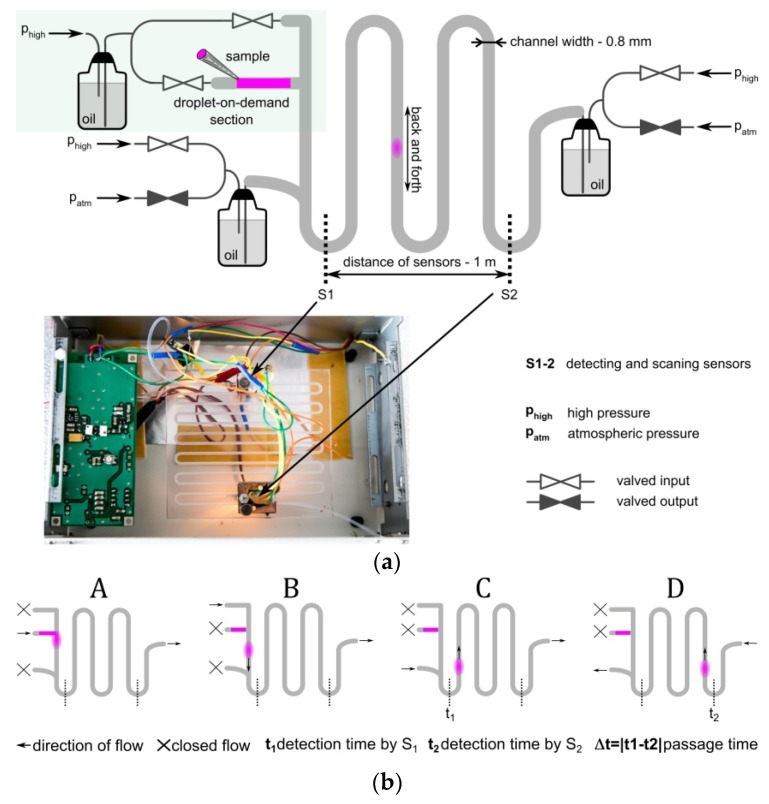
(**a**) Schematic of the microfluidic device used in the experiments. The photograph shows the central part of the system with marked sensors (see also Figure S1). (**b**) **A**–**D** show the procedure of the droplet generation in a (A,B) droplet-on-demand section and the (C,D) cycling of a droplet back and forth between sensors.

The central part of the measurement system consisted of a 1.2 m (i.e., the distance between the valves that governed the circulation of the droplets) Teflon Fluorinated Ethylene Propylene (FEP) tubing (I.D. 0.8 mm, O.D. 1.6 mm, BOLA, Bohlender GmbH, Grünsfeld, Germany) (Figure 1). The tubing was fixed in a milled 5 mm thick polycarbonate plate (Macroclear, Bayer, Germany) which acted as a rigid support. The flow of the continuous phase was induced by a stable pressure difference (100 ± 0.01 mbar) between two tanks that were located before and after the circulation control valves (V165, equipped with Z070D coils, Sirai, Italy). All of the aqueous samples were injected into the chip in the droplet-on-demand section, directly from sterile syringes and were stored inside the tube. Next, the flow of oil pushed the sample into the main tubing using the droplet-on-demand technique, which provided an accurate control of the droplet size (Figure 1b). Then, the generated droplet was cycled back and forth between the sensors in order to measure the passage time that was required for the calculation of viscosity. Herein, the volume of the drop was 2.4 μL, which corresponded to a length that was equal to the six internal diameters of the tube. The measurement system was immersed in distilled water.

We controlled the pressure that was applied to the oil reservoirs using triple series manual pressure regulators (Bosch Rexroth PR1-RGP, Lohr, Germany) and monitored the pressure using digital manometers (AZ 82100, AZ Instruments, Taichung City, Taiwan). The generation and detection of droplets were aided by an edge-detection algorithm [35] that tracked the process of the microdroplet formation and controlled the volume with an accuracy of ~0.1%. The necessary image feedback was provided by a digital camera (uEye UI-3140CP-C-HQ Rev.2, IDS, Mannheim, Germany).

The detectors (sensors) contained an LED light source (HMIB-44WY-TR7, Huey Jann Electronics, Taipei, Taiwan) and an optical photodetector (TSL257, Taos, New Mexico, NM, USA). Both of the elements were powered from a low noise source (build on LT3042, Analog Devices, Norwood, MA, USA) and the level of the voltage noise was 50 μV (root mean square of voltage). The proper signal processing was provided by low noise amplifiers LT1028 (Linear Technology, Milpitas, CA, USA). The whole system enabled the monitoring of the density of the cells in the microdroplet and the absorbance was recorded at 600 nm.

The temperature was stabilised to within 0.1 °C in a thermostatic styrofoam box with the use of custom-made electronics.

## 3. Results

### 3.1. Calibration of the Measurement System

**Figure 2:**
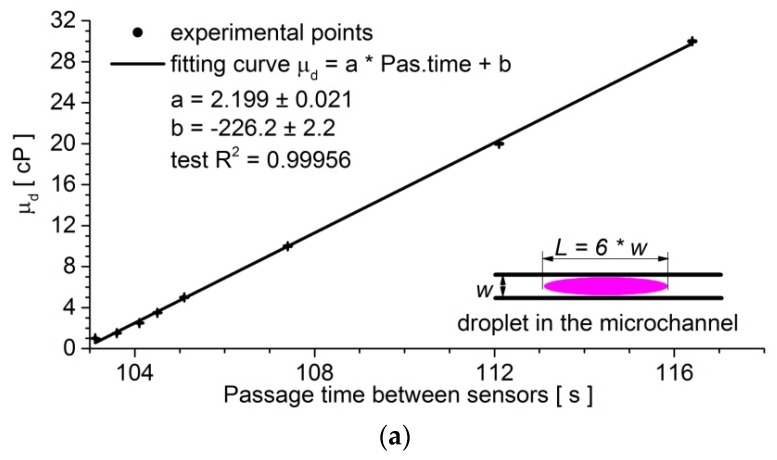

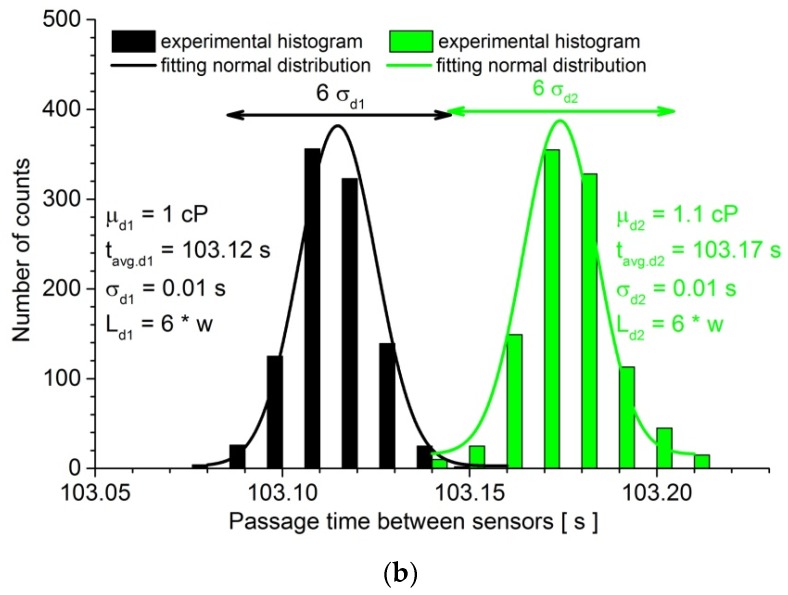
(**a**) The plot of the relationship between the viscosity of a droplet (*µ_d_*) and the time of passage between the sensors. The standard deviations of the passage time and droplet viscosity over ten samples were marked on the graph. (**b**) The chart shows the distribution of passage time of a droplet between the sensors. The experiment was repeated for the sample that consisted of 1000 independent droplets with the same viscosity (*μ_d_*_1_ = 1 cP and *μ_d_*_2_ = 1.1 cP, water-glycerin solutions). The normal distribution was fitted to both histograms. The experiments were performed for ∆*p_continuous phase_* = 100 mbar in temperature T = 37 °C.

The calibration of the viscosity measurement was performed using various mixtures of water and glycerol with known viscosities, under a predefined pressure difference between oil tanks (100 mbar) and temperature conditions (37 °C). The measurements of the transition times in the microfluidic device were conducted for a 2.4-μL droplet that was travelling between the sensors. The obtained calibration plot demonstrated a recorded linear relationship between the viscosity of a droplet and its time of flow between the sensors, with test *R*^2^ = 0.99956 (Figure 2a). The viscosity increased from 1 cP to 30 cP for passage times from ca. 103 s to ca. 116 s, respectively. It indicated that the drops streamed slower upon the increase of viscosity. In Figure 2b, the Gaussian distributions of the viscosity measurements for the two different solutions have been presented. It could be concluded that, under these conditions (∆*p_continuous phase_* = 100 mbar, T = 37 °C), the resolution of the system was 0.05 cP.

**Figure 3:**
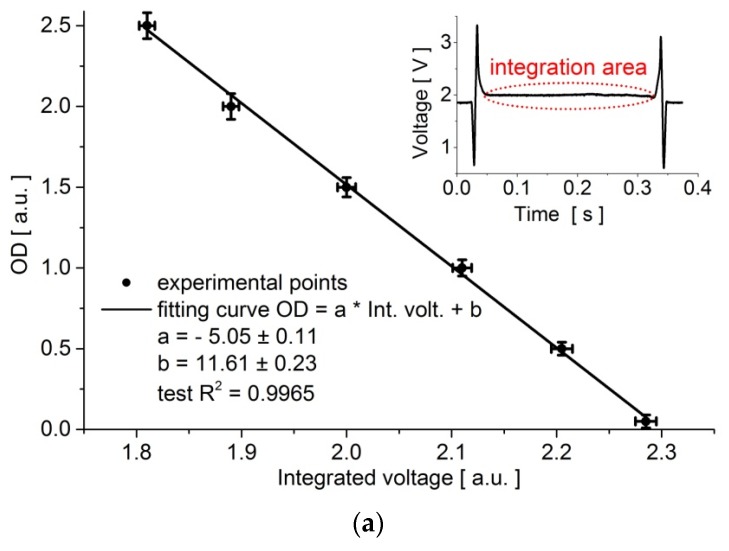

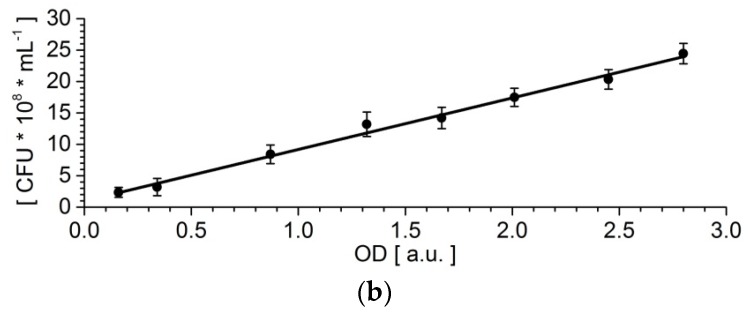
(**a**) The relation between the optical density (OD) of a droplet that was generated in the system and the integrated voltage signal that was recorded by the sensor during the droplet passage. The inset (the dependence of sensor voltage vs. time) shows the area of integration for a passing droplet, where the beginning and the end of the droplet are marked with sharp voltage spikes. (**b**) The correlation between the number of colony-forming units (CFU) per mL and optical density (OD). He colonies were plate-counted after the dilutions of the bacterial culture media. The standard deviations over ten samples were marked on the graphs. The experiments were performed for ∆*p_continuous phase_* = 100 mbar in temperature T = 37 °C.

We also compared the optical density (OD) of a generated droplet that contained a bacterial medium with the integrated voltage signal, which was recorded by the sensor during the droplet passage. The OD of the respective solutions was measured using a spectrometer (CCS200/M, Thorlabs, Newton, NJ, USA) before injection to the storage tubing. It was observed that this calibration function was also linear in a whole voltage range from 1.8 to 2.3 a.u. (Figure 3a). The optical density of a droplet that was generated in this system decreased steadily with voltage from OD_1_ = 2.5 a.u. to OD_2_ = 0.05 a.u., as illustrated in Figure 3a.

To verify that the value of OD measured in the droplet is proportional to the number of bacteria, we conducted the bacterial culture test using the dilution method for the selected OD values. In Figure 3b, a well-defined linear dependence observed for the number of bacterial cells in a sample vs. OD is presented.

### 3.2. Viscosity Measurements during Bacterial Culture Growth

**Figure 4:**
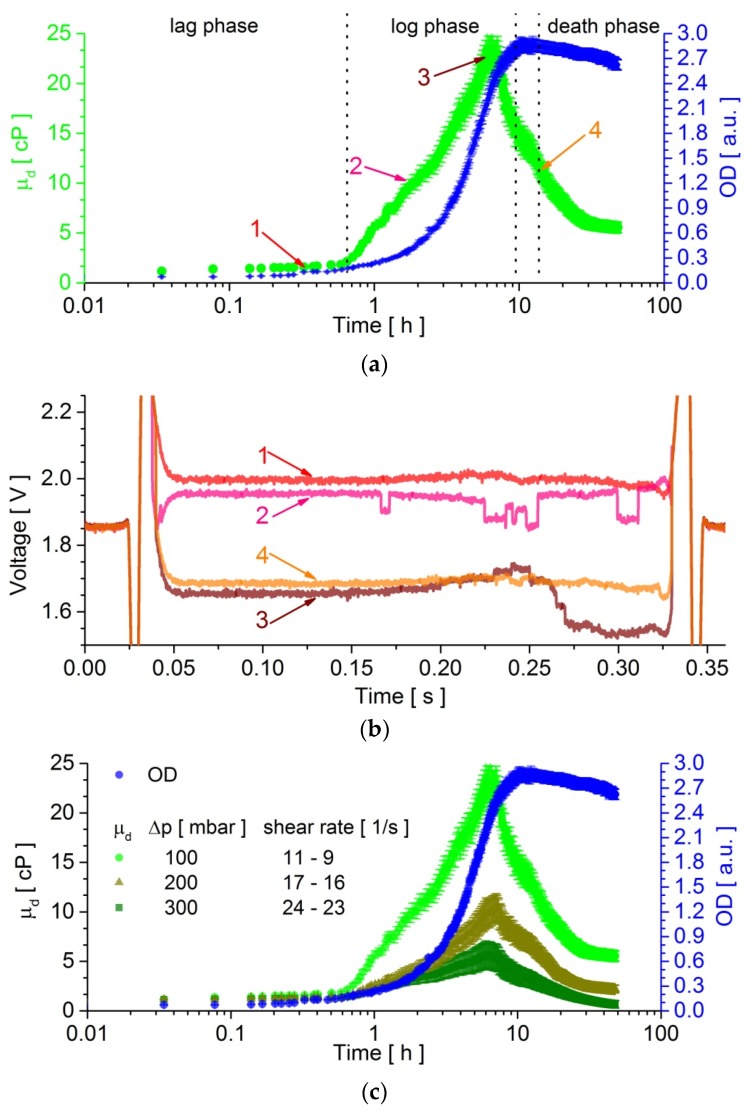
Time evolution of the droplet viscosity (*µ_d_*) and optical density (OD) for a circulating droplet filled with bacterial culture for (**a**) ∆*p_continuous phase_* = 100 mbar and (**c**) ∆*p_continuous phase_* = 200 and 300 mbar. The standard deviations of the OD and droplet viscosity over ten samples were marked on the graphs (**a**,**c**). The OD curve was reproducible for all three of the cases (**c**). The experiments were performed in temperature T = 37 °C. (**b**) The voltage signals (1–4) that were recorded by the sensors during the flow of a droplet under them for various viscosities of a sample (for ∆*p_continuous phase_* = 100 mbar). Corresponding viscosity points were labeled at (**a**). The front of the droplet is on the left side of the graph.

Using the presented system, we measured, simultaneously and in real-time manner, the viscosity and the OD of the droplet that contained a growing bacteria culture, over the incubation period of 48 h. The experiment was conducted ten times in order to obtain solid curves (the relative standard deviation was marked in the graphs of Figure 4a,c). The changes of the temporal variation of the viscosity of a medium that contained bacteria with an increasing colony abundance were presented in Figure 4a. It has been shown that in the lag phase, the detected changes in viscosity are negligible. During the log phase, a strong increase of viscosity from 1.83 ± 0.38 cP to 23.31 ± 1.86 cP was observed. After ca. 6 h, the viscosity of a medium that contained bacteria decreased sharply and this decrease was also continued in the death phase, down to ca. 5.5 cP. The recorded changes in the OD of a single droplet that contained bacterial culture is shown in Figure 4a. It was seen that the optical density increased from the lag phase 0.05 a.u. through the log phase to OD_max_ = 2.83 ± 0.07 a.u., and then slightly decreased in the death phase. To verify that the observed viscosity and OD had changed solely because of the bacterial growth-related processes, we also recorded the viscosity and OD for a negative control droplet that contained only the nutrient. The obtained results are presented in Figure S2. It was visible that the values of the viscosity and OD were invariant with time, within the experimental error. The measurements for the droplet that contained only the nutrient were performed between the measurements of the droplet with bacteria. Herein, for the pressure difference, 100 mbar was applied to the system and the shear rate equalled zero across the droplet in its middle and reached the maximum value at the interface, ca. 11 s^−1^ (Ca = 2 × 10^−4^ for *μ_d_* = 1.7 cP) and ca. 9 s^−1^ (Ca = 1.76 × 10^−4^, *μ_d_* = 24 cP), which was based on the calculation made by Coulliette et al. [36] and Erb et al. [37].

We also measured the bacteria culture for the applied pressure difference 200 and 300 mbar, in order to observe changes in the viscosity of the bacteria culture for higher shear rates. The obtained outcomes are plotted in Figure 4c. The graph shows that for shear ranges between 17–16 s^−1^ (for 200 mbar) and 24–23 s^−1^ (for 300 mbar) while the viscosity of bacteria reaches smaller maximal value respectively ca. 10 cP and 5.5 cP than ca. 23 cP for the shear range 11–9 s^−1^ (for 100 mbar). All three of the curves had similar characters for the specific growth phases of bacteria. The OD curves were reproducible for droplets with various shear rates.

Moreover, we also analysed the recorded voltage signal from the optical sensors when a droplet that contained the bacteria culture was flowing under them (Figure 4b). For the small viscosity of sample (i.e., circa 1.5 cP), the scanned optical signal along the droplet was uniform (Figure 4b-1). When the viscosity of the bacterial culture increased up to ~10 cP, the recorded curve along the droplet exhibited abrupt changes, which indicated a greater adsorption of light in the place of a lower voltage signal (Figure 4b-2). It could be interpreted that in such places, the abrupt reduction of the signal contained a more significant concentration of bacteria, that is, complex aggregates of bacteria. A further increase of the viscosity (~23 cP) allowed us to observe only one large conglomerate of bacteria that was located at the back of the droplet (Figure 4b-3). After reaching the maximum viscosity of the sample, when its viscosity had decreased (~11 cP), it turned out that the conglomerate of bacteria disappeared, and the signal from the sample was again quite homogeneous (Figure 4b-4). The observed changes in the signal of the optical density along the droplet allowed for correlating the increase and decrease of the viscosity of the bacterial culture with the bacterial structures that formed inside the droplet during the growth of the microorganisms.

In the measured samples, despite the large OD, the viscosity decreased after reaching the maximum. That suggested a drop of living bacteria fraction, despite the large OD signal. However, spread tests (five agar plates on each value OD: 2.7, 2.6, and 2.5) from the droplets that were mixed with fresh LB broth confirmed that the large population of bacteria was consistent with the OD reading.

### 3.3. The Verification of Cross-Contamination of the Continious Phase

We verified whether the presence of microorganisms in droplets led to the contamination of the inside of the tubing (biofilm formation) or the oil phase (crossing of the water-oil interface). After the incubation and viscosity measurements, the continuous phase was removed from the tanks and oil was mixed with fresh LB broth and incubated for three days. Next, the medium was spread on ten LB agar plates so as to conduct the smear test. In this experiment, no growth of bacteria was observed after one week of incubation. Moreover, the droplets with a negative control were also spread on ten LB agar plates. Furthermore, in this case, no growth of microorganisms was observed (one week observation).

## 4. Discussion

The recorded outcomes showed that the viscosity of the medium with a bacterial culture increased with the increasing optical density up to the inflexion point on the optical density curve (Figure 4a), which was the lag and most of the log phase. According to Leal’s group [23,24], the increase of the viscosity of the bacterial culture was influenced by the growth of the number of bacteria, by self-organisation of the bacteria into structures, and their collective interactions [38]. It was also evident in our outcomes (Figure 4c) that the change of a shear rate caused the alteration in the recorded viscosity of the bacteria culture. This indicated that the appearance of complex aggregates that were created by microorganisms depended on the shear stress inside the droplet. The smaller the shear rate was, the more complex the structure of bacteria that was created. The creation of conglomerates of bacteria was also visible in the signal coming from the optical sensors, which were scanning the droplet along while it was flowing in their sector of the device (Figure 4b). At the inflexion point of the OD curve, the viscosity of the bacteria suspension reached a maximum and then decreased, despite the continuing increase of OD, still in the log phase. In the stationary and death phases, when the OD begans to decrease, the viscosity of the microorganisms’ culture decreased asymptotically to ca. 5.5 cP after 48 h of culturing. The recorded changes of the viscosity for the cultured bacteria in the stationary and death phases significantly deviated from the results that have been presented by Portela et al. [24]. They measured, with the use of a standard rotational rheometer, the viscosity of *Escherichia coli* DH5α, (i.e., the same strain we have measured in this work) and found that it to be invariant in these phases. The differences that were encountered between these experiments was likely to be because of the different measurement methods that were adopted. In our experiments, the culture of bacteria was enclosed in a droplet that was continuously flowing, which was carried by the hydrofluoroether oil. The HFE oil family had the ability to dissolve gases to high concentrations and exchange them with the second phase across the interface, so as to amplify the bacterial growth [39,40,41]. In turn, in the Portela et al. [24] experiments, the bacteria culture was placed in a rheometer in a plate-plate geometry with a constant shear rate of 1 × 10^−1^ s^−1^, which used a solvent to avoid evaporation.

The viscosity of an active suspension of *Escherichia coli* bacteria was determined experimentally in the dilute and semidilute regime, using a Y-shaped microfluidic channel by Gachelin et al. [42]. Their work concerned the flow of a bacteria medium as a continuous phase. However, they transferred the bacteria, after washing from a rich medium, to the motility medium so as to consider them as pushers from a physical point of view. Herein, we considered the growth of bacteria in a rich LB medium. Therefore, the active mixing of bacteria [43], cooperative interaction between microorganism [28,44], and a collective motion at both a micro and macro scale [45,46,47] must have had an impact on the recorded growth rates of the bacterial culture viscosity. The decrease in bacterial culture viscosity in the later stages of the experiments could be associated with nutrients depletion, alternative secreted metabolites, motility change, and/or limited access to oxygen. Douarche et al. [48] showed that motile bacteria strains, for example *Escherichia coli*, could change their motility characteristics in few minutes and eventually stop swimming as a result of the lack of oxygen, which explained the change in the viscosity of bacterial culture. The complexity of the problem have suggested further research on this subject.

## 5. Conclusions

We have demonstrated that the proposed novel rheometer system enables simultaneous measurements of the droplet viscosity and its absorbance (optical density) in real time. It uses very small quantities (ca. 2 µL) of the sample, which is especially important for biological samples, such as the pathogenic cultures. The proposed method can also be utilized for systems that require gas exchange for normal growth or functioning [40]. The resolution of the measurement system can be increased either by increasing the size of the droplet or by reducing its speed in the microchannel (see Figure S3). Importantly, the viscosity of the dispersed phase, which is higher than the viscosity of the continuous phase, always increases the hydrodynamic resistance of the system. This conclusion opens new possibilities in the area of blood screening, such as the evaluation of the impact of drugs on viscosity and the clotting of blood. These capabilities of the method that have been described here provide new opportunities for studying biological systems.

## Supplementary Materials

The following are available online at http://www.mdpi.com/2072-666X/9/5/251/s1, Figure S1: Photographs of the experimental system. Figure S2: Time evolution of the droplet viscosity (*µ_d_*) and optical density (OD) for a circulating droplet filled with bare nutrient without bacteria. The standard deviations of OD and droplet viscosity over ten samples were marked on the graph. The experiments were performed for ∆*p_continuous phase_* = 100 mbar in temperature T = 37 °C. Figure S3: The charts show the distribution of passage time of a droplet between sensors. The experiments were repeated for the sample consisting of 1000 independent droplets with the same viscosity. The normal distribution was fitted to histograms. The experiments were performed in temperature T = 37 °C. (a) the volume of the droplets was 2.4 μL, which corresponded to a length that was equal to the six internal diameters (*w*) of the tube *L_d_*_1_ = *L_d_*_2_ = 6·*w*, ∆*p_continuous phase_* = 100 mbar, tested droplets viscosities: *μ_d_*_1_ = 1 cP and *μ_d_*_2_ = 1.1 cP, (b) the volume of the droplets was 2.4 μL, which corresponded to a length that was equal to the six internal diameters (*w*) of the tube *L_d_*_1_ = *L_d_*_2_ = 6·*w*, ∆*p_continuous phase_* = 45 mbar, tested droplets viscosities: *μ_d_*_1_ = 1 cP and *μ_d_*_2_ = 1.1 cP, (c) the volume of the droplets was 2.4 μL, which corresponds to a length that was equal to the six internal diameters (*w*) of the tube *L_d_*_1_ = 6·*w* and 4.8 μL, which corresponds to a length that was equal to the twelve internal diameters (*w*) of the tube *L_d_*_1_ = 12·*w*, ∆*p_continuous phase_* = 100 mbar, tested droplets viscosity: *μ_d_*_1_ = *μ_d_*_2_ = 1 cP.
