# Supplementary material for: Simultaneous Measurement of Viscosity and Optical Density of Bacterial Growth and Death in a Microdroplet
Source: Micromachines (Basel). 2018 May 21;9(5):251. doi: 10.3390/mi9050251 (PMC6187717; doi:10.3390/mi9050251)
Supplement: Supplementary file 1 [file micromachines-09-00251-s001.pdf]

Supporting information

Simultaneous Measurement of Viscosity and Optical Density of Bacterial Growth and Death in a Microdroplet

Karolina Sklodowska [1,2], Pawel R. Debski [1], Jacek A. Michalski [3], Piotr M. Korczyk [4], Miroslaw Dolata [5], Miroslaw Zajac [1] and Slawomir Jakiela [1,*]

1 Department of Biophysics, Warsaw University of Life Sciences, 159 Nowoursynowska Street, 02776 Warsaw, Poland; karolina_sklodowska@sggw.pl (K.S.); pawel_debski@sggw.pl (P.R.D.); miroslaw_zajac@sggw.pl (M.Z.)
2 Department of Plant Genetics, Breeding and Biotechnology, Warsaw University of Life Sciences, 159 Nowoursynowska Street, 02776 Warsaw, Poland
3 Faculty of Civil Engineering, Mechanics and Petrochemistry, Warsaw University of Technology, 17 Lukasiewicza Street, 09400 Plock, Poland; jacek.michalski@pw.edu.pl
4 Institute of Fundamental Technological Research, Polish Academy of Sciences, Pawinskiego 5B, 02-106 Warsaw, Poland; piotr.korczyk@ippt.pan.pl
5 Department of Econophysics and Physics Application, Warsaw University of Life Sciences, 159 Nowoursynowska Street, 02776 Warsaw, Poland; miroslaw_dolata@sggw.pl
* Correspondence: slawomir_jakiela@sggw.pl; Tel.: +48-22-593-8626

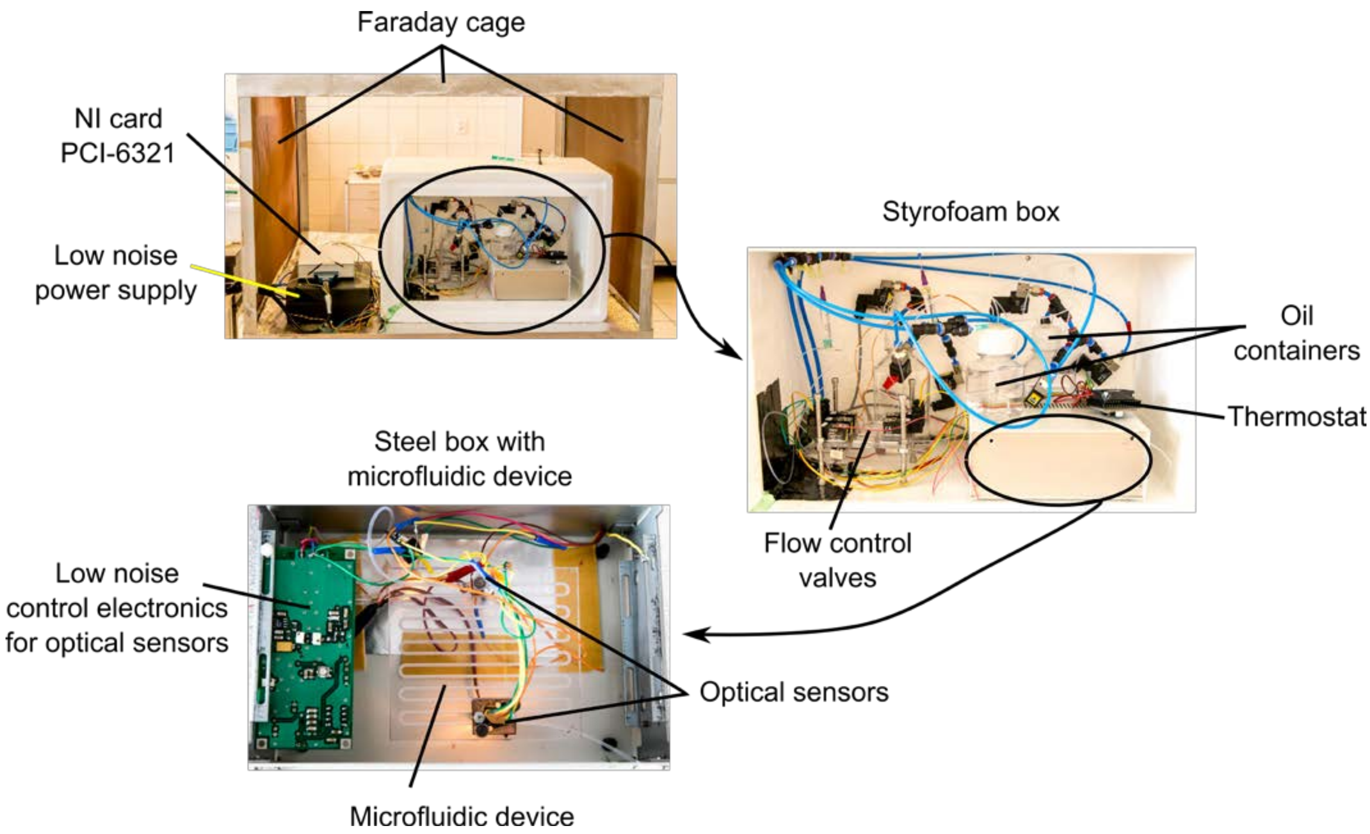

Figure S1. Photographs of the experimental system.

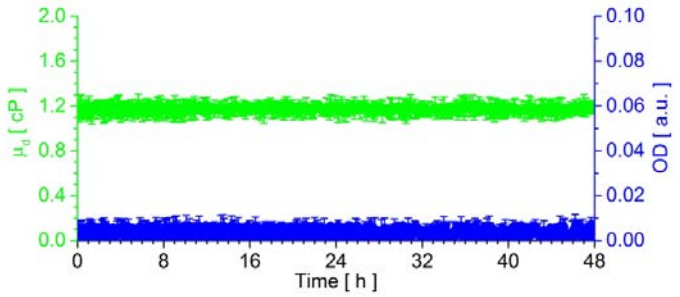

Figure S2. Time evolution of the droplet viscosity (μ_d) and optical density (OD) for a circulating droplet filled with bare nutrient without bacteria. The standard deviations of OD and droplet viscosity over ten samples were marked on the graph. The experiments were performed for $\Delta p_{continuous\ phase}$ = 100 mbar in temperature T = 37 °C.

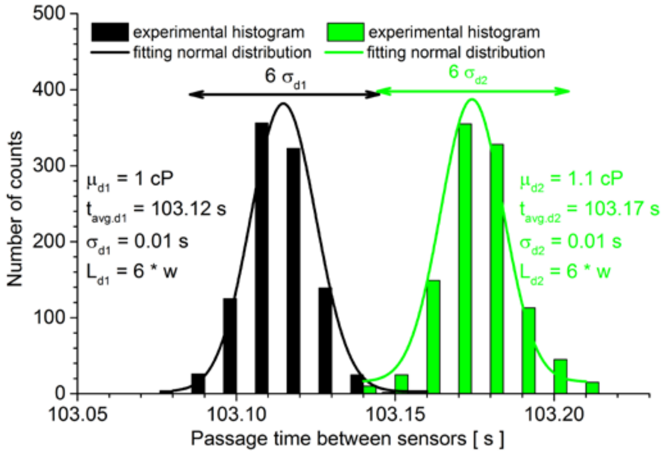

(**a**)

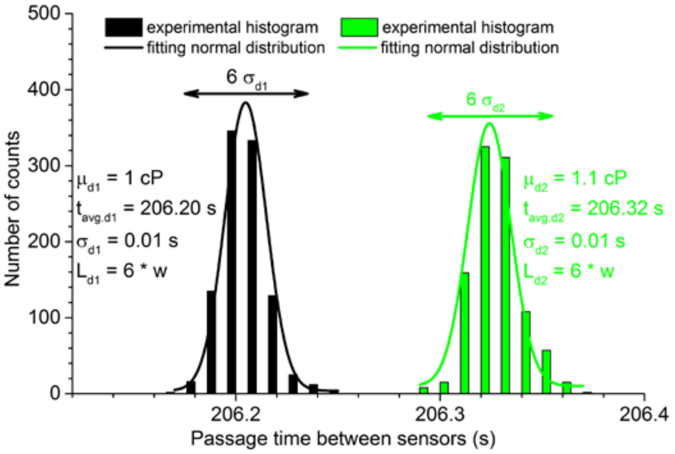

(**b**)

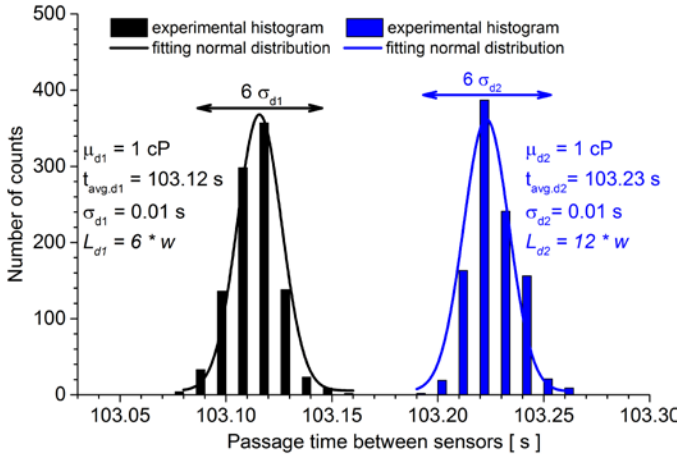

(**c**)

Figure S3. The charts show the distribution of passage time of a droplet between sensors. The experiments were repeated for the sample consisting of 1000 independent droplets with the same viscosity. The normal distribution was fitted to histograms. The experiments were performed in temperature T = 37 °C. **(a)** the volume of the droplets was 2.4 μL, which corresponded to a length that was equal to the six internal diameters (w) of the tube $L_{d1} = L_{d2} = 6 \cdot w$, $\Delta p_{continuous\ phase}$ = 100 mbar, tested droplets viscosities: μ_{d1} = 1 cP and μ_{d2} = 1.1 cP, **(b)** the volume of the droplets was 2.4 μL, which corresponded to a length that was equal to the six internal diameters (w) of the tube $L_{d1} = L_{d2} = 6 \cdot w$, $\Delta p_{continuous\ phase}$ = 45 mbar, tested droplets viscosities: μ_{d1} = 1 cP and μ_{d2} = 1.1 cP, **(c)** the volume of the droplets was 2.4 μL, which corresponds to a length that was equal to the six internal diameters (w) of the tube $L_{d1} = 6 \cdot w$ and *4.8* μL, which corresponds to a length that was equal to the twelve internal diameters (w) of the tube $L_{d1} = 12 \cdot w$, $\Delta p_{continuous\ phase}$ = 100 mbar, tested droplets viscosity: $\mu_{d1} = \mu_{d2}$ = 1 cP.
